# Six-Port Based Interferometry for Precise Radar and Sensing Applications

**DOI:** 10.3390/s16101556

**Published:** 2016-09-22

**Authors:** Alexander Koelpin, Fabian Lurz, Sarah Linz, Sebastian Mann, Christoph Will, Stefan Lindner

**Affiliations:** Institute for Electronics Engineering, Friedrich-Alexander University of Erlangen-Nuremberg, Erlangen D-91058, Germany; fabian.lurz@fau.de (F.L.); sarah.linz@fau.de (S.L.); sebastian.mann@fau.de (S.M.); christoph.will@fau.de (C.W.); stefan.lindner@fau.de (S.L.)

**Keywords:** six-port, interferometer, radar, industrial sensing, microwave sensor

## Abstract

Microwave technology plays a more important role in modern industrial sensing applications. Pushed by the significant progress in monolithic microwave integrated circuit technology over the past decades, complex sensing systems operating in the microwave and even millimeter-wave range are available for reasonable costs combined with exquisite performance. In the context of industrial sensing, this stimulates new approaches for metrology based on microwave technology. An old measurement principle nearly forgotten over the years has recently gained more and more attention in both academia and industry: the six-port interferometer. This paper reviews the basic concept, investigates promising applications in remote, as well as contact-based sensing and compares the system with state-of-the-art metrology. The significant advantages will be discussed just as the limitations of the six-port architecture. Particular attention will be paid to impairment effects and non-ideal behavior, as well as compensation and linearization concepts. It will be shown that in application fields, like remote distance sensing, precise alignment measurements, as well as interferometrically-evaluated mechanical strain analysis, the six-port architecture delivers extraordinary measurement results combined with high measurement data update rates for reasonable system costs. This makes the six-port architecture a promising candidate for industrial metrology.

## 1. Introduction

Industry and manufacturing technology is experiencing a revolution these days. The demand for products with a high degree of individuality leading to a huge variety of configurations is increasing more and more. Automated production with the help of robots repeating precisely the steps they have been taught will be too inflexible for this modern manufacturing approach. The vision is to change from automated production towards intelligent production by adding cyber physical functionality to the process steps, i.e., the complete manufacturing process will be self-organizing and flexibly adaptive to the current conditions. In this context, the digitalization of all production processes interacting and exchanging information among each other, as well as with the work pieces is self-evident. However, in the current discussion, another missing component for realizing the vision of cyber physical manufacturing comes more and more into focus: the ability of the production machinery to react automatically on changing preconditions in their environment. Robots for example have to detect obstacles in their motion trajectory and find an unobstructed path to their target position while preventing all potential collisions. Just as well, precise remote metrology for the context sensitive and adaptive control of the robot is essential.

In this framework, remote and contact-free sensing of distances, angles or motions plays a key role and pushes new sensing approaches, which complement existing sensor technologies to cover all potential measurement tasks arising from the mentioned vision. State-of-the-art technologies, like ultrasonic or optical distance measurement units, are able to cover a certain part of the required metrology applications. However, in harsh industrial environments or for high precision measurements combined with update rates in the kilohertz domain, new concepts have to be found.

Based on the significant progress in monolithic microwave integrated circuit (MMIC) technology during the last few decades, remote sensing systems using radio frequency (RF) approaches show compelling performance combined with reasonable costs. Especially in the microwave domain, radio detection and ranging (RADAR) evolved into promising metrology for industrial sensing applications [1]. A benefit of microwave sensors is that they can be used simultaneously for both obstacle detection and precise distance and motion control. However, classic radar architectures may exhibit also drawbacks by some means or another. This motivates the investigation of an alternative microwave sensing concept: the six-port microwave interferometer. This publication will give a review of the six-port approach for industrial sensing, demonstrates the use for classic industrial metrology applications and discusses also the limitations of this architecture.

Section 2 will give an introduction in the general concept of the six-port architecture and its building blocks. In Section 3, the extensions of the basic six-port system to cope with industrial distance, angle and mechanical strain sensing will be presented. The non-ideal behavior of real six-port interferometer hardware will be discussed in Section 4, and linearization, as well as compensation techniques will be shown to reduce the influence of the these impairments. Section 5 is dedicated to a review of the pros and cons of six-port interferometry for industrial measurement applications and compares this concept to state-of-the-art metrology. Section 6 finally concludes this paper.

## 2. Six-Port Architecture

The six-port architecture is nowadays a quite uncommon technology. For this reason, the following section will review the general concept and functionality.

### 2.1. Basic Principle

The six-port technology in general has been known since the first half of the 20th century, but was first intensely promoted in the 1970s by Glenn F. Engen and Cletus A. Hoer. Their publication “Application of an Arbitrary 6-Port Junction to Power-Measurement Problems” [2] is regarded as the beginning of contemporary six-port technology. The authors describe a setup to enhance common directional scalar power measurement systems using two power detectors with additional phase information by adding another two detectors. The proposed concept forms one port of a reflectometer or vector network analyzer. Consequently, their ongoing work is dedicated to improving the system for building reflectometers [3] for metrology applications. Within 28 years from the first beginnings, the system has undertaken an evolution up to a two-port vector network analyzer including full calibration [4].

Besides vector network analysis, six-port theory made its way into other topics [5]. Examples are material characterization and microwave spectroscopy [6,7]. Some years ago, a near-field scanning microscope has been proposed based on the multi-port structure [8]. Even in communication, the six-port concept has been introduced [9,10,11,12].

About 20 years after the beginning of six-port technology for reflectometer applications, the first publications that proposed this concept as a radar system can be found [13,14,15,16,17]. Compared to all other topics, this aspect has been treated rather sporadically and only from a very basic point of view. The radar application has been picked up nowadays, and detailed investigations for both system aspects and building blocks have been conducted [18,19,20].

The name “six-port” is directly derived from the structure itself: it features two input and four output ports, thus six ports. The basic concept of six-port technology can be summarized by phase controlled superposition of two input signals $S_{1}$ and $S_{2}$ that are superimposed under four different relative and static phase shifts. The resulting four sum signals can be observed at the respective output ports of the structure. These six ports exhibiting all of the same frequency in the microwave domain are characteristic for the six-port concept [21].

**Figure 1:**
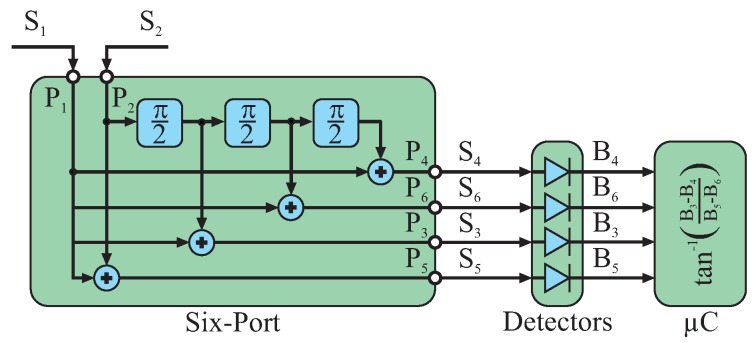
The two input signals $S_{1}$ and $S_{2}$ are superimposed inside the six-port structure under four different static phase shifts $\Phi_{S}$ that are multiples of π/2 .

The most common and efficient way for the six-port’s internal superposition of $S_{1}$ and $S_{2}$ is to choose integer multiples of π/2 for the static phase shifts, e.g., $\Phi_{S,3}=0\pi$ , $\Phi_{S,5}=\pi /2$ , $\Phi_{S,4}=\pi$ and $\Phi_{S,6}=3\pi /2$ (Figure 1). These superpositions will lead to constructive or destructive interference depending on the relative phases of the two input signals. Therefore, the power values of these sum signals $S_{3}$ to $S_{6}$ are a measure for the relative phase $\Delta \varphi +\Phi_{S,3...6}$ in between $S_{1}$ and $S_{2}$ . The input signals $S_{1}$ and $S_{2}$ are connected to port numbers $P_{1}$ and $P_{2}$ of the six-port; the output signals $S_{3}$ to $S_{6}$ can be observed at ports $P_{3}$ to $P_{6}$ .

By analyzing the four different power values of $S_{3}$ to $S_{6}$ , all information of the input signals can be retrieved. When considering the static relative phase shifts for the six-port’s internal superposition, two subsets can be formed: $\Phi_{S,3}=0\pi$ and $\Phi_{S,4}=\pi$ result in the first couple; $\Phi_{S,5}=\pi /2$ and $\Phi_{S,6}=3\pi /2$ are the second pair. Each couple will represent a specific signal and a counter-phased copy of it by a rotation of *π*. Between both couples, there is an additional rotation of π/2 . Therefore, the four different output signals of the six-port $S_{3}$ to $S_{6}$ can be interpreted as a differential representation of a complex valued number $\underline{Z}$ or *I/Q* (in phase/quadrature) signal. The couple $\Phi_{S}\left(0\pi ;\pi \right)$ forms the real or in phase (I) part, the other one $\Phi_{S}\left(\pi /2;3\pi /2\right)$ the imaginary or quadrature (Q) part.

Regarding $S_{1}$ as the reference signal and applying the actual $\Phi_{S,3..6}$ of the presented six-port structure, these relations read as shown in Equations (1)–(4).

(1) $$\begin{array}{ccc} S_{3} & = & 0.5(S_{1}+jS_{2}) \end{array}$$

(2) $$\begin{array}{ccc} S_{4} & = & 0.5(S_{1}-jS_{2}) \end{array}$$

(3) $$\begin{array}{ccc} S_{5} & = & 0.5(S_{1}+S_{2}) \end{array}$$

(4) $$\begin{array}{ccc} S_{6} & = & 0.5(S_{1}-S_{2}) \end{array}$$

To reconstruct the relative dependency of the input signals $S_{1}$ and $S_{2}$ from $S_{3}$ to $S_{6}$ , the power values of the latter have to be measured. Assuming a common characteristic impedance for the system, there is a quadratic dependency between the power of a signal and its amplitude. For simple signal processing, a conversion stage will do a power to voltage transformation converting $S_{3}$ to $S_{6}$ to baseband voltages $B_{3}$ to $B_{6}$ . This down-conversion step can be mathematically and ideally described by the squared Euclidean norm ${\left|.\right|}^{2}$ of the signals $S_{3}$ to $S_{6}$ as shown in Equations (5)–(8).

(5) $$\begin{array}{ccc} B_{3} & = & {\left|S_{3}\right|}^{2}={\left|0.5(S_{1}+jS_{2})\right|}^{2} \end{array}$$

(6) $$\begin{array}{ccc} B_{4} & = & {\left|S_{4}\right|}^{2}={\left|0.5(S_{1}-jS_{2})\right|}^{2} \end{array}$$

(7) $$\begin{array}{ccc} B_{5} & = & {\left|S_{5}\right|}^{2}={\left|0.5(S_{1}+S_{2})\right|}^{2} \end{array}$$

(8) $$\begin{array}{ccc} B_{6} & = & {\left|S_{6}\right|}^{2}={\left|0.5(S_{1}-S_{2})\right|}^{2} \end{array}$$

As already stated for the microwave output signals $S_{3}$ to $S_{6}$ , the baseband voltages $B_{3}$ to $B_{6}$ can be interpreted as a complex valued number in differential representation as shown in Equation (9).

(9)Z_=(B5−B6)+j(B3−B4)(10)ℜ{Z_}=B5−B6(11)ℑ{Z_}=B3−B4

In communication, digital modulation concepts are very popular, which often use a complex valued representation for the information to be transmitted. One common principle is quadrature amplitude modulation (QAM) or *I/Q* modulation, where the data are represented by symbols distributed over the complex valued number plane. For retrieving the data from the modulated signal, the real and imaginary parts have to be determined. It can be directly concluded from Equations (10) and (11) that a receiver based on six-port technology is capable of demodulated such *I/Q* modulated signals. Only two baseband voltages, respectively, have to be subtracted from each other leading, to real part I and imaginary part Q. Renato Bosisio [22] and Ke Wu [23] are well regarded for their investigations of six-port receivers for digital communications. This application as a communication receiver is worldwide the main focus of most six-port-related research groups [24,25,26,27].

Since the complex valued number $\underline{Z}$ can be represented by a vector, it is easy to derive the argument of this vector from Equations (10) and (11). The resulting argument of $\underline{Z}$ in Equation (13) is equivalent to the original phase difference Δφ between the two input signals $S_{1}$ and $S_{2}$ .

(12)Δφ=arg(Z_)=arctanℑ(Z_)ℜ(Z_)(13)=arctanB3−B4B5−B6

All metrology concepts based on six-port architecture will use its functionality to represent the information contained in the relative variation between a reference and the measured signal as a complex valued number or directly as a vector.

### 2.2. Building Blocks

The previous section introduced the six-port as a tool for superimposing two signals under four different relative phase shifts. In general, such a system is called an interferometer. Depending on the operating frequency, different practical realizations are suitable. The essential building blocks, the name giving six-port structure and the power detectors, can be complemented by further circuitry, e.g., low noise amplifiers (LNA), automatic gain controls (AGC) or phase shifters.

Depending on the operation frequency, cost requirements or system performance, all building blocks can be implemented in a hybrid way by mounting commercial MMICor custom assemblies of basic lumped components on a microwave-capable printed circuit board (PCB). Alternatively, custom-designed dedicated MMIC integration of the total six-port interferometer is also feasible. Especially for frequencies beyond 100 GHz, the latter solution is very interesting to ensure good overall performance.

#### 2.2.1. Basic Building Blocks

##### Six-Port Structure

**Figure 2:**
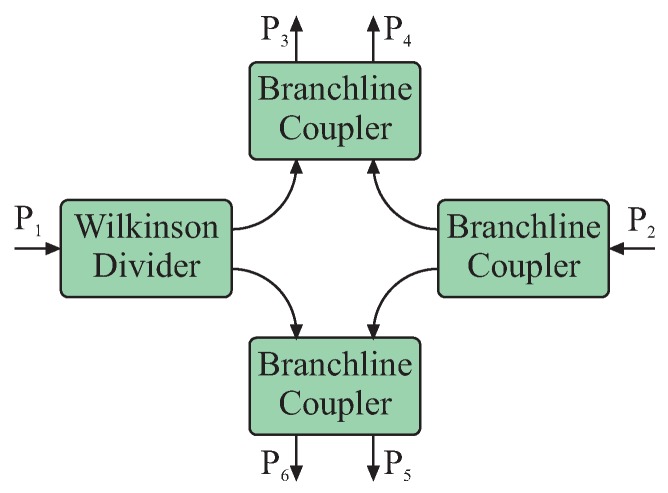
Passive structure of a six-port interferometer formed by three Branchline quadrature hybrid couplers and one Wilkinson power divider [28].

Within the scope of this publication, six-port systems for frequencies in the Industrial, Scientific and Medical (ISM) bands for 2.45 GHz, 24 GHz and 61 GHz will be presented. For these frequencies, both input signals can be passively superimposed by coupler structures, e.g., quadrature hybrid couplers and Wilkinson power splitters. This coupler circuitry forms the six-port structure and ensures well-defined phase relations between both input and all four output signals. In general, several different coupler topologies are feasible for the passive six-port structure [5], while all systems presented in this work are based on the same architecture formed by three quadrature hybrid couplers and one Wilkinson power divider (Figure 2). The advantage of this topology is the almost identical power relationship for both input signals at any output port and nearly ideal static phase relationships with multiples of π/2 . This relaxes the requirements on signal conditioning and dynamic range maximization.

##### Power Detector

**Figure 3:**
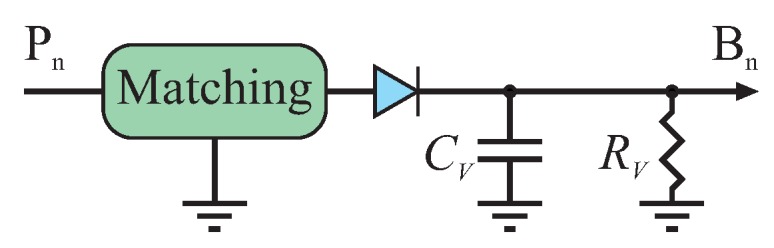
Diode power detector for down-converting the six-port’s output signals.

Due to the superposition of the input signals inside the passive six-port structure, the relative power and phase between these signals lead to characteristic power scaling of the output signals in the microwave domain. For basic interferometer functionality, the power of these four output signals has to be converted to proportional baseband representations. The easiest and most common way is using diode power detectors at the four output ports of the six-port, as shown in Figure 3, which transfer the respective microwave power to an equivalent baseband voltage. The diodes are operated in their so-called square law region, where the quadratic term of their Taylor series dominates the characteristic. If this square law region is exceeded, the power detection will show distortions that will degrade the interferometer’s linearity if not compensated. For a static scenario with constant input signals, the four baseband voltages will be DC.

##### Baseband Processing

Using the proposed six-port structure will result in four baseband voltages at the diode power detectors’ output ports, which can be interpreted as a differential complex valued (*I/Q*) voltage signal. Therefore, the reconstruction of the desired phase difference Δφ between the input signals of the system can be conducted in the analog or the digital domain by subtracting the positive and negative differential signal parts from each other, respectively. The analog subtraction reduces the number of parts for the analog baseband signal conditioning, as well as analog-to-digital converters (ADC) from four to two and eliminates some offset errors, but reduces flexibility for system calibration. Therefore, for the systems presented in this paper, all four baseband signals are digitized, and the subtraction is carried out in the digital domain. This will directly result in the *I/Q* signal. If the subtraction is conducted in the analog domain, the resulting *I/Q* signal should be digitized to calculate the argument of this complex valued *I/Q* vector. Furthermore, this algorithm shows low implementation complexity, since the utilized arc tangent function values can be stored in a look up table (LUT) to speed up the processing time.

#### 2.2.2. Advanced Building Blocks

Besides these basic building blocks, most interferometric systems have to be supplemented by additional hardware to cope with non-ideal effects, environmental influences or complex system requirements. The choice of additional modules strongly depends on the measurement task and the demands in accuracy arising from the application. In Section 3, it will be explained for the different applications which of these additional building blocks might be useful for most cases of the respective measurement task. It has to be kept in mind that phase or amplitude impairments may be induced by some of these building blocks that may have to be compensated in the digital domain.

##### Low Noise Amplifier

For nearly any system, at least one additional low noise amplifier (LNA) should be added as the first component for the input branch of the six-port’s receive path. This ensures better noise performance and power equalization between the reference signal coupled from the transmit path and the receive signal. The best interferometric performance will be reached if both signals are nearly on the same power level.

##### Variable Attenuator

**Figure 4:**
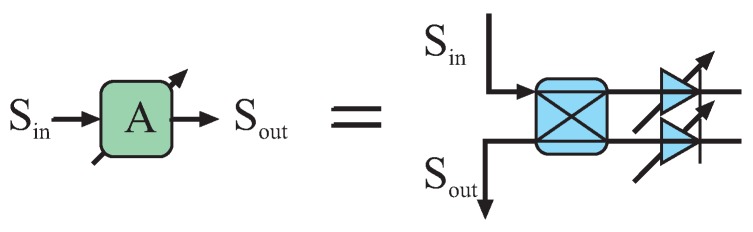
Symbol and schematic of a reflection-type variable attenuator.

The power of the reference signal at the six-port input can be statically defined by the coupling coefficient of the directional coupler in the transmit path. For calibration purposes of the power detectors’ characteristics, this coupling coefficient can be chosen quite high, followed by a variable attenuator to adaptively sweep the power of this strong reference signal. In the microwave domain, attenuators can be easily realized in a reflection-type configuration, which is shown in Figure 4.

##### Automatic Gain Control

**Figure 5:**
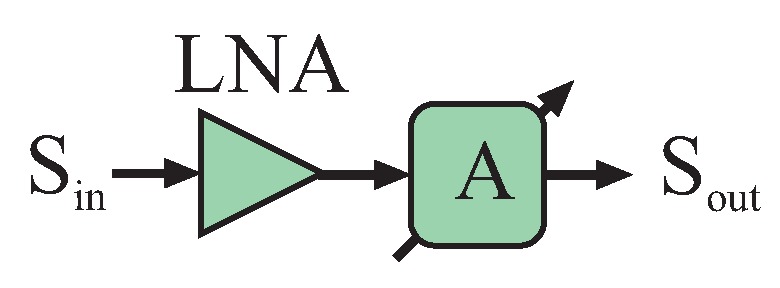
An automatic gain control (AGC) in the microwave domain can be formed by a low noise amplifier (LNA) followed by a variable attenuator.

If the dynamic range of the receive signal exceeds the dynamic range of the power detector and for enhancing the sensitivity of the interferometer, an automatic gain control (AGC) can be implemented in the receive path at the six-port structure’s input port. For this reason, LNA and variable attenuator stages are cascaded (Figure 5). Attention has to be paid to ensure stability for this microwave AGC.

Besides an AGC in the microwave domain, also a baseband AGC should be implemented for scaling the baseband DC voltages to the maximum dynamic range of the ADC. This baseband AGC can be simply constructed, e.g., by operational amplifiers with digitally-adjustable potentiometers.

##### Offset Compensation Circuitry

**Figure 6:**
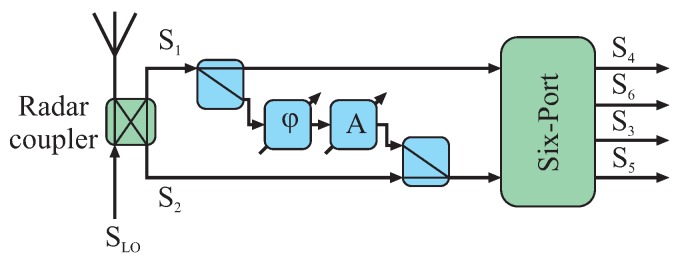
Offset compensation structure (in blue) to enhance the isolation between both six-port input ports.

Due to non-ideal effects, portions of the transmit signal may also be present at the receive path of the six-port, severely limiting total system performance. Special offset compensation circuitries may be used to superimpose a portion of the transmit signal to the receive path in counter phase and with equal power as the parasitic transmit signal component. Extensive compensation will occur if the gain and phase of the compensation circuitry are chosen correctly. This building block can be constructed by a variable phase shifter followed by a variable attenuator and the respective microwave couplers. The blue components in Figure 6 exemplarily show such a compensation in between the input ports of the six-port structure.

##### Microwave Switch

**Figure 7:**
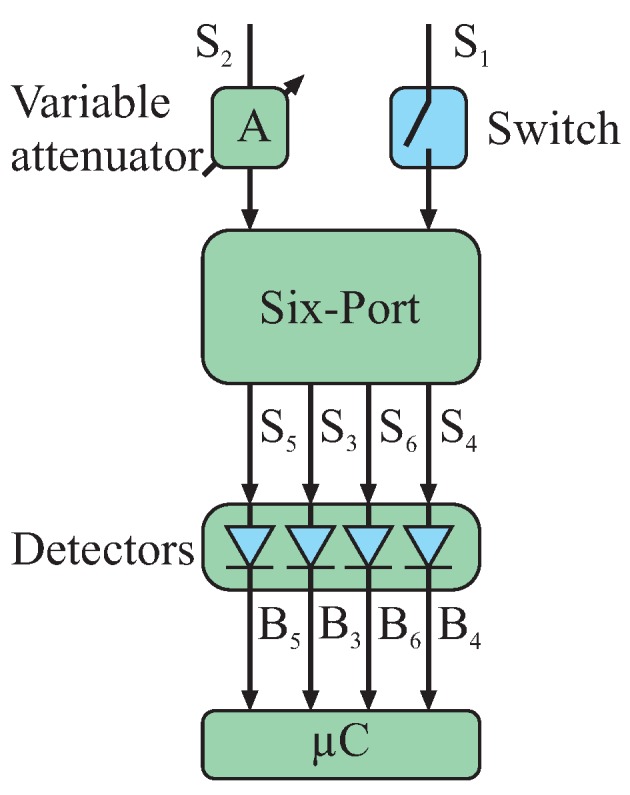
Exemplary circuitry for deriving the characteristics of all diode detectors by switching off one input signal and sweeping the power of the other continuous wave (CW) input signal.

For the calibration purposes of the system, additional switches can be used to shut down one of the input signals, respectively, and thereby measuring the signal power distributed from the active input port of the six-port to the four detector branches. Furthermore, for deriving the diode detectors’ characteristics a shutdown switch for the receive path will be used in conjunction with the variable attenuator in the reference path (Figure 7).

## 3. Six-Port in Metrology

Six-port technology has always been a niche topic. There are only a few research groups in the world investigating this receiver architecture. Most of them focus on the six-port as a receiver for *I/Q* data [10,24,29] or investigate network analysis setups [30,31,32]. However, already from the early beginnings, there have been sporadic publications using the six-port concept as a radar receiver. Often, only the analog microwave front-end or laboratory setups for the evaluation of the basic feasibility have been presented. These publications demonstrate the suitability of this architecture for distance sensing [13,33]. Another dominant part of these presented works substitutes the homo- or hetero-dyne down-conversion mixer stages by a six-port receiver utilizing frequency modulated continuous wave (FMCW) as the signal modulation scheme [17,34]. Furthermore, combinations of the common FMCW radar concept and six-port continuous wave (CW) interferometry have been proposed [14]. Furthermore, six-port radars showed their usability for Doppler and velocity measurements [15,35].

This paper is focused on the usage of six-port technology for metrology in the industrial framework of making robots cognitive and adaptable to changes in their environment. The measurement tasks that can be addressed in this context by the six-port interferometer are distance, alignment, i.e., angle of arrival detection, and mechanical strain analysis.

### 3.1. Distance

**Figure 8:**
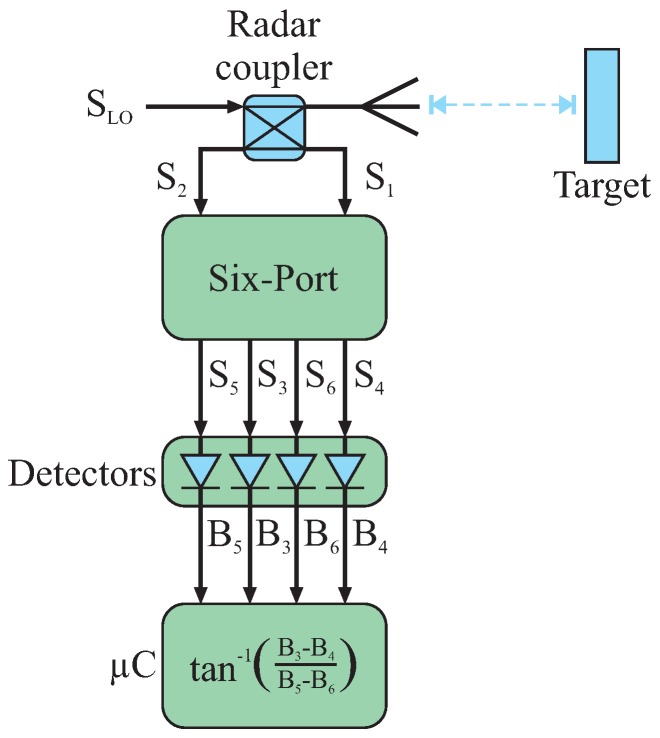
Six-port system for interferometric distance detection.

Six-port interferometry is able to cope with all measurement applications where the value under investigation can be mapped to a relative phase measurement. For distance measurements, the change in target position will directly vary the path length between the sensor and target, hence the relative phase of a received signal scattered by the target in comparison to its transmitted signal [36,37]. Figure 8 complements the basic six-port building blocks to a mono-static radar system. In addition to the passive six-port structure, the diode detectors and the baseband processing the system comprise an RF oscillator generating the transmit CW signal $S_{LO}$ . A portion $S_{1}$ of the oscillator signal is coupled by a radar coupler, e.g., a quadrature hybrid, to one input port of the six-port as a phase reference; the rest of $S_{LO}$ is transmitted to an antenna and radiated. The radiated signal is scattered by the target and received by the same antenna (for a mono-static system). For high performance systems, the offset compensation circuity introduced may be added. The receive signal is coupled as signal $S_{2}$ to the other input port of the six-port by the radar coupler and may be amplified by an LNA. Assuming the target to be situated in the far-field area of the antenna, a distance variation Δd between the antenna’s phase center and the target will directly result in a change of the relative phase shift between the reference and receive signal proportional to Δd . The displacement Δd is calculated by means of Equation (14) using Equation (13) and the wavelength of the reference signal *λ* [38]:

(14) $$\begin{array}{ccc} \Delta d & = & \lambda \frac{\arg(\underline{Z})}{4\pi } \end{array}$$

Due to the ambiguity in the obtained phase shift Δφ by 2π , Δd is ambiguous, as well. Therefore, an unambiguous displacement can only be detected within the range of λ/2 . For larger displacements, relative measurements can be realized by unwrapping the phase or counting the running periods and adding the respective multiples of 2π as the offset to the measured phase value.

**Figure 9:**
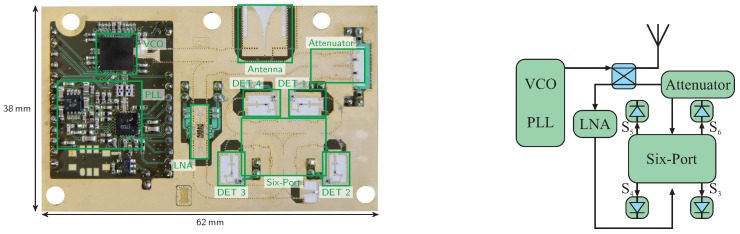
Photo and schematic of a 61-GHz six-port radar front-end realized in substrate-integrated-waveguide (SIW) technology.

Figure 9 shows a photo and the schematic of a 61-GHz mono-static sensor front-end in substrate-integrated-waveguide (SIW) technology based on the six-port radar principle. By reason of the employed SIW technology, the six-port structure shows excellent performance in a broad frequency range [38,39]. The sensor is similar to the system presented in [40]; however, it features an additional LNA and is fully integrated on a single PCB. The signal generation is realized by commercial MMIC and operates in the range from 57 GHz to 64 GHz. The LNA in the receive path is bonded to the PCB using ribbon bonds to ensure good matching over a broad bandwidth.

**Figure 10:**
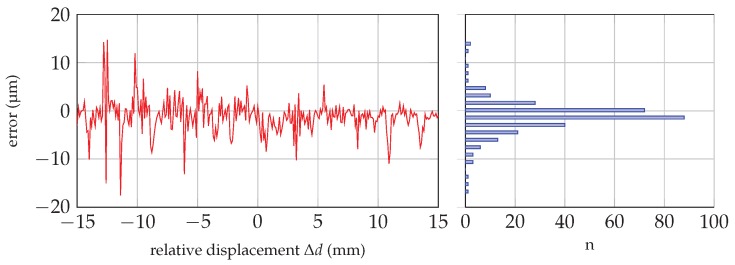
Measurement error over reference value Δd and the absolute quantity error distribution.

Intended to be used as a near-field sensor, the system has been tested over a range of 30 mm, whereas the system’s zero-point has been chosen to be located at a distance of d=25 mm in front of the antenna. For evaluation measurements, a planar metallic target with dimensions of 100 mm × 80 mm has been moved in the range of Δd=±15 mm around the zero-point using a linear stage with an optical position encoder of 0.5-µm resolution as the reference. The six-port radar system has been operated in continuous wave mode at a frequency of 60 GHz; therefore, all acquired measurement values are referenced to the zero-point. As the target is applied in the near-field range, measurement data are influenced by inter-reflection effects. These influences have a stronger impact, if the distance between the target and antenna decreases. Figure 10 shows the obtained linearized distance error of a single measurement (sample rate of 1 kHz) as a function of the acquired reference value of the linear position encoder on the left-hand side. The absolute error distribution of the displayed measurement curve is plotted as a bar plot, additionally. The error distribution has a bell-shaped Gaussian curve; however, the obtained error is bigger for smaller *d* values, though for negative relative displacement values.

### 3.2. Angle of Arrival

Besides distance evaluation, also other geometrical relations can be measured by six-port interferometry. Garcia et al. presented in [41] a six-port-based angle of arrival (AoA) detection system for an electromagnetic wave impinging on the receiving antennas. The proposed system uses the high phase resolution of the six-port receiver by replacing the multiplicative homodyne receivers for a conventional AoA detection array. Each receive path consists of a dedicated six-port receiver with a common local oscillator (LO) distribution network in between. The AoA calculation and post-processing can be conducted as for active mixing AoA setups.

However, there is an even more sophisticated concept for six-port-based AoA detection. The major difference for the described approach is that the AoA system can be reduced to only one single six-port receiver without any LO circuitry [42]. Furthermore, the AoA can be directly derived by low complexity equations. This approach will be discussed in the following.

**Figure 11:**
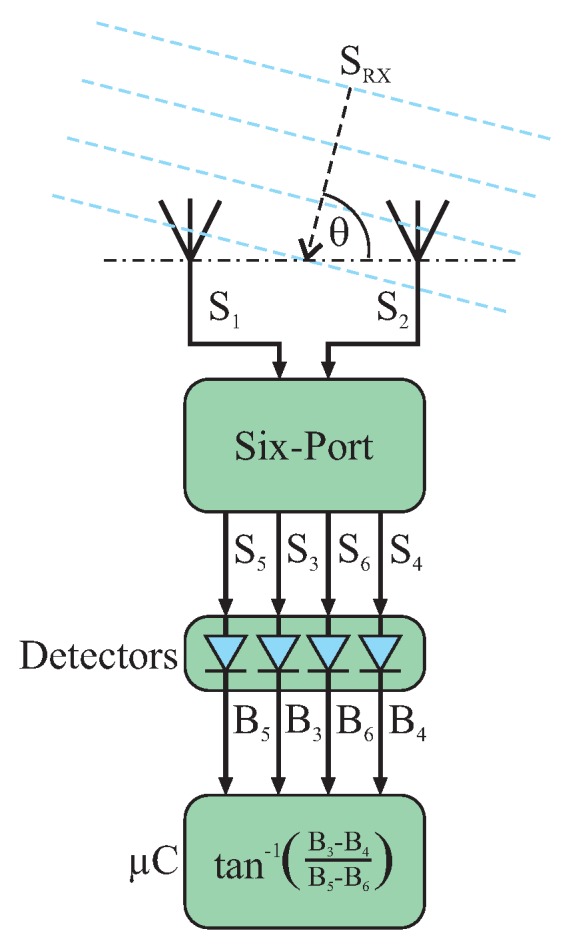
Six-port system for interferometric angle of arrival detection.

Key is the mapping of the AoA to the six-port based phase difference measurement Δφ from its baseband voltages $B_{3}$ to $B_{6}$ . This equation is the same as for any six-port application. Both input ports of the six-port structure are directly connected to receive antennas whereupon the connecting line of their antenna centers form the reference line for the AoA detection. As shown in the schematic of Figure 11, the impinging wave $S_{RX}$ in the far field of the transmitter can be approximated by a plane wave. In this case, the AoA *θ* can be derived from Δφ in between both receive antennas by trigonometrical dependencies and read as shown in Equation (15) while knowing the wavelength *λ* of the impinging wave and the receive antenna distance *a*. Details for this relationship can be found in [43].

(15) $$\begin{array}{ccc} \theta & = & \sin^{-1}\left(\frac{\lambda \Delta \varphi }{2\pi a}\right) \end{array}$$

**Figure 12:**
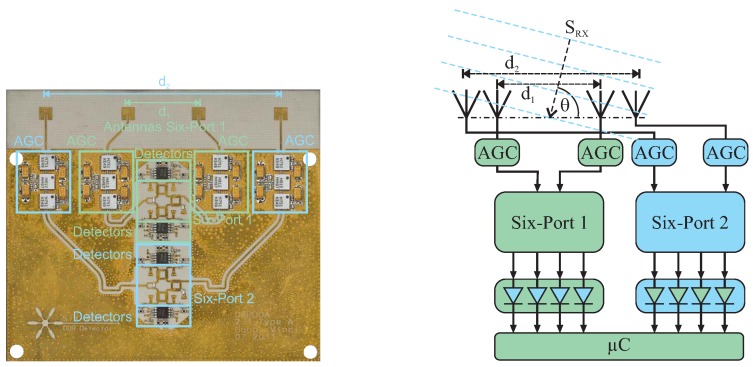
Photo and schematic of a dual six-port AoA system combining high angular resolution with a wide observation range.

This AoA analysis approach features extremely low complexity, especially for short distances between the transmitter and six-port module, where in some cases, no LNA will be required due to the intrinsically low power requirement at the detectors for ensuring the square law. This makes the six-port-based AoA well suited for industrial applications, e.g., the alignment of automotive radar sensors with respect to the thrust vector of a car during maintenance in automotive workshops. Since only the passive six-port circuitry and diodes for the detectors are used, this approach is easy to scale in frequency, e.g., for millimeter wave operation. In Figure 12, a photo and the schematic of a 24-GHz setup combining two separate AoA detection systems are shown. The advantage of this dual six-port approach is that a high observation range combined with high angular resolution in the milli-degree range can be achieved, as is discussed in [43].

One has to keep in mind that the input signals to the six-port will be superimposed and directly down-converted without any chance to separate more than one transmitter. However, for most industrial AoA-based alignment applications, the scenario is well known and can be limited to only one signal source in the main lobe of the receive antennas, as is the case for the automotive radar alignment.

**Figure 13:**
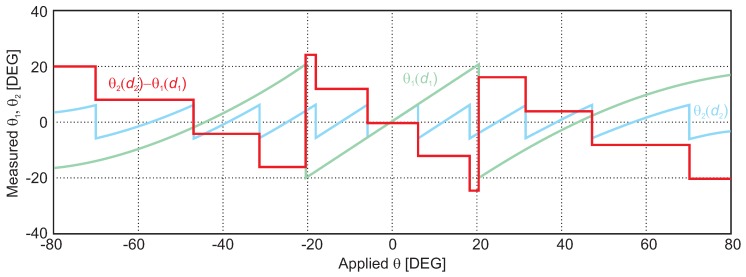
System simulation results for AoA analysis for two six-port interferometers shown in Figure 12 featuring different antenna distances and resulting in calculated angles $\theta_{1}$ (in green) and $\theta_{2}$ (in blue), respectively. The difference between both graphs (red curve) leads to a step-like graph indicating the respective period for ambiguity elimination (system measurements presented in [42]).

An exemplary alignment measurement is shown in Figure 13: a 24-GHz CW signal having −30 dB output power and placed in a distance of 2 m is transmitted via a 20-dBi horn antenna to the six-port AoA measurement system shown in Figure 11. The relative alignment between the transmit antenna and the six-port receiver is swept by a rotation stage over a range of $\theta =\pm 80^{\circ }$ . The wave, i.e., the phase front arriving at both six-port receive antennas, can be regarded as approximately planar at the position of the receiver. Therefore, the linear sweep in alignment angle results in a linear phase sweep. The presented AoA detector features two separate six-port systems having different receive antenna distances. Since this antenna distance *d* defines the phase periodicity with respect to the angle deviation resulting from Equation (15), different graphs can be measured for the six-ports, respectively. This feature is used to solve the ambiguity issue of 2π by subtracting both values for identical measurement steps leading to values characteristic for the actual period *n* (plotted as the red graph). This offset of (n·2π)−1 is added to the more precise AoA value of the six-port system having a smaller ambiguity free range, but higher angular resolution (i.e., Six-port 2). In conclusion, by using two systems featuring different antenna distances, the ambiguity free range of AoA can be enlarged while ensuring the high angular resolution of the more precise system. By the presented methodology extremely precise alignment tools for industrial metrology can be built.

### 3.3. Mechanical Strain

Resonant surface acoustic wave (SAW) devices are nowadays used for various wireless sensing applications, especially in harsh and industrial environments. A change of physical parameters, like temperature or mechanical strain, directly leads to a change of the sensor’s resonance frequency [44]. As these are purely passive devices, a reader system is required to interrogate the sensor. To this day, several different reader concepts [45,46,47] for resonant SAW sensors have emerged. The latest systems measure the sensor’s self-resonance frequency directly by transmitting an excitation signal and processing the response signal of the resonator that contains the desired information, typically by means of a fast Fourier transform (FFT) [48]. Recently, six-port-based SAW sensing has been shown as a promising alternative to conventional FFT-based reader systems [49]. Instead of calculating a time-consuming FFT on an embedded device, the sensor’s response signal is processed already in the analog domain based on the concept of instantaneous frequency measurement [50]: by using a delay line of known length, the challenge of a precise frequency determination is mapped to a phase measurement that can be quickly evaluated by the six-port network. This leads to very short measurement times and low hardware costs.

**Figure 14:**
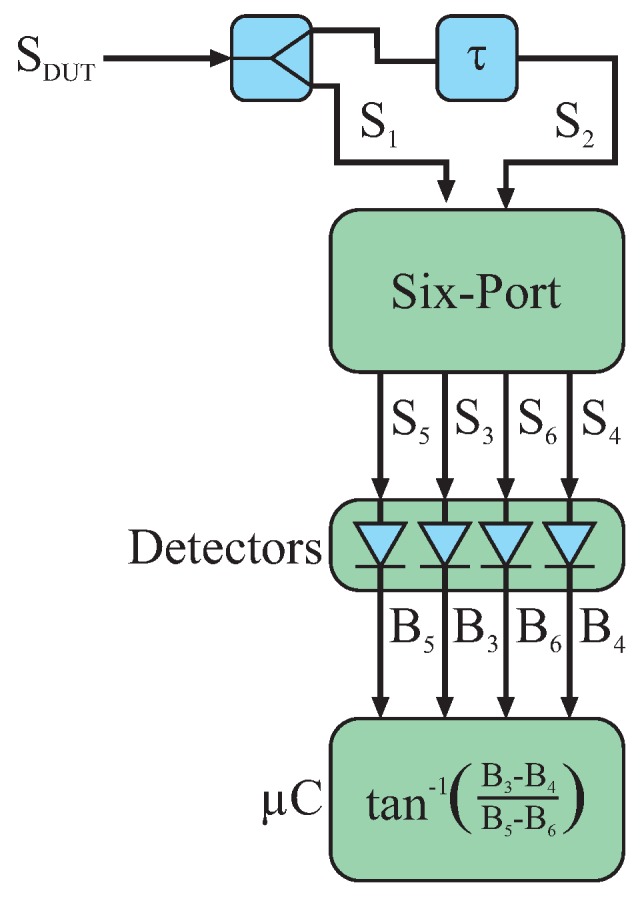
Six-port system for interferometric frequency detection.

The system concept of six-port-based interferometric frequency detection is depicted in Figure 14.

The response signal of the sensor $S_{\mathrm{DUT}}$ is split into two portions. One part $S_{1}$ is fed directly to the first input port of the six-port while the other part $S_{2}$ passes through a delay line with a time delay of *τ* before reaching the second input port. This leads to a frequency-dependent relative phase shift Δφ of:

(16) Δφ=2πfτ.

With the knowledge of *τ* and the measured phase difference Δφ , the frequency $\tilde{f}$ can finally be calculated:

(17) $$\tilde{f}=\frac{\Delta \varphi }{2\pi \cdot \tau }\;.$$

However, care has to be taken as these calculations become ambiguous when the effective length of the delay line becomes longer than the wavelength of the highest frequency to be determined. Generally, the system has only a limited unambiguous bandwidth of:

(18) $$f_{B}=\frac{1}{\tau }\;.$$

For the application of mechanical strain sensing, this is not really a limitation, as the maximum frequency deviation of the SAW sensors is typically in the range of one MHz or even below. However, when a more broadband solution is required, multiple parallel systems with various delay line lengths can be combined [50].

**Figure 15:**
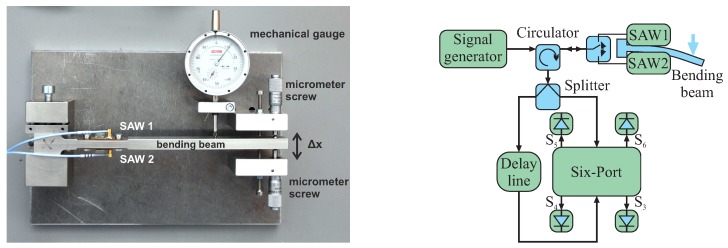
Photo and schematic of the measurement setup with a mechanical bending beam featuring two surface acoustic wave (SAW) resonators in a differential configuration.

**Figure 16:**
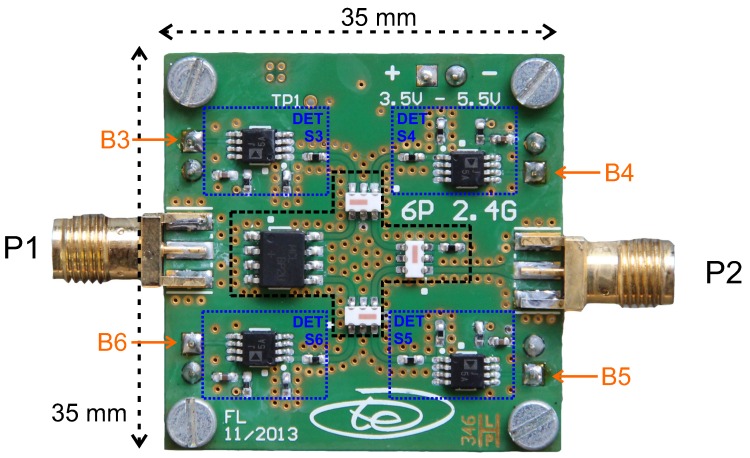
Photo of the 2.4-GHz six-port interferometer.

**Figure 17:**
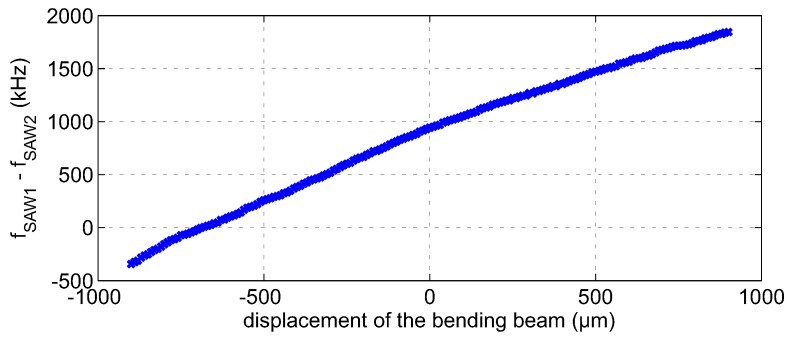
Measurement results for a differential mechanical strain analysis using two 2.4-GHz SAW resonators.

It has been shown that a differential measurement setup considerably improves the sensitivity, as well as the accuracy of the measurement setup [51]. Figure 15 depicts a photo and schematic of the measurement setup featuring a mechanical bending beam with two mounted SAW resonators in a differential configuration. A signal generator is used as the excitation source. The excitation pulse passes a circulator and a single pole double throw switch that selects one of the resonators. After quickly shutting off the excitation, the response signal of the stimulated sensor is split into two equal parts by a power splitter. One part is directly fed into the six-port interferometer, while the other one has to pass the delay line, leading to the relative phase shift (Equation (16)) between the input ports. The delay line is currently realized as a 10-m RG58-U SMAcable. However, more compact solutions based on high permittivity dielectrics or SAW delay lines are possible. A photo of the used 2.4-GHz six-port interferometer is depicted in Figure 16. The design is based on lumped components leading to a very compact realization with PCB dimensions of 35 mm × 35 mm [52]. Figure 17 depicts the measurement results for a differential mechanical strain analysis. In total, 181 equidistantly-spaced deflections of the bending beam have been measured, interrogating the SAW resonators with the six-port interferometer. The peak-to-peak frequency difference of the two resonators is approximately $\Delta f_{pp}$ = 2.18 MHz. With no strain applied, Δf is about 940 kHz, and the standard deviation has been determined to be $\sigma_{f}$ = 21.9 kHz. This leads to a standard deviation of approximately 1% related to the sensor’s full scale range. The curve exhibits a slight non-linearity mainly due to non-ideal mounting of the resonators, as well as component imperfections of the delay line and the six-port system. Especially the latter ones can be reduced with proper in situ linearization concepts.

## 4. Non-Ideal Behavior of the Six-Port

**Figure 18:**
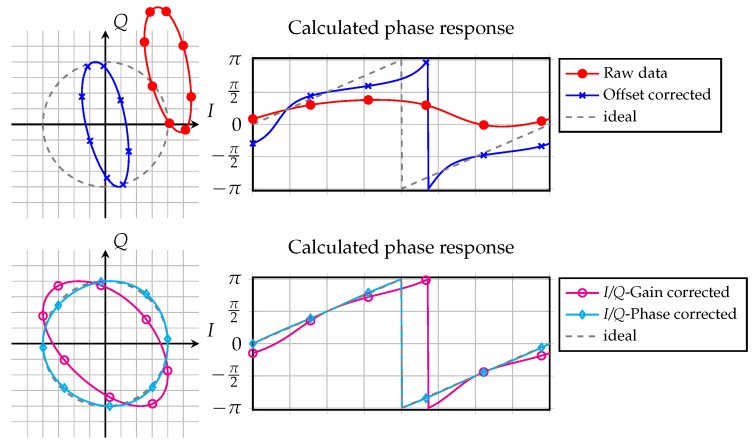
Raw measurement data and calibration steps in in phase/quadrature (*I/Q*) and the phase domain.

Non-ideal effects of the front-end may lead in some cases to severe degradation of the ideal six-port behavior. One major problem is voltage offsets at the detectors’ baseband outputs resulting from different effects, which are depicted as raw data in Figure 18. It stands to reason that it is not possible to use the raw measurement data to calculate the phase. Due to the huge offsets, the phase response did not cover the complete phase range from 0 to 2π . Therefore, the phase error, which can be calculated as the difference between the phase responses and the dashed ideal case, is very large. Even in the case of removed offset errors, there is still a strong non-linear behavior within the phase responses. This can further be suppressed by canceling the I/Q -impairments [53], which is shown in the lower plots. The resulting deviation from the ideal case is due to non-linearities and has to be addressed by additional linearization steps.

The sources causing these errors, as well as possible approaches to remove them will be clarified in the next sections.

### 4.1. Static Offset

The biggest influence on the non-ideal behavior of the six-port interferometer is generated by static reflections of the TX RF signal inside the front-end. The reasons for this phenomenon are manifold, e.g., parasitic coupling from the synthesizer’s high gain output signal to other front-end structures due to housing reflections or limited shielding, limited isolation between the TX and the RX path of the radar coupler, any mismatch between components, the antenna, vias and random reflections [54]. All of these parasitic signal components will interfere with each other and lead to one sum signal featuring the power integration of all portions, the same frequency as the TX signal, and an equivalent phase depending on the weighted contribution of each signal. In general, disregarding possible temperature drifts or aging, this parasitic signal is static over time and will result in different DC offsets at the power detectors’ baseband output ports. Since the power of the interference signal depends on the relative phases between the parasitic and the TX signals, the relative amplitudes and also phases at the four detectors’ RF input ports are differing. In the worst case, the resulting detector input signals do not even show the intended relationship of multiples of π/2 . This deforms the unit circle as the intended complex representation and leads to an offset ellipse as depicted in the upper left plot of Figure 18. Regarding the corresponding phase curves in the upper right plot, the reconstruction of the desired measurement value by calculating the argument of a complex valued number as shown in Equation (13) is nearly impossible.

Therefore, the major system design goal must be to minimize the static parasitic reflection signals coupled from the TX path to the RX side of the six-port. The easiest way to reduce these signal components is to use bi-static front-end configurations with a careful isolation between the TX and RX antenna. As there is by definition no common path between the TX and RX signal for a bi-static front-end, static reflections by mismatch and as a consequence parasitically-induced DC offsets at the diode detectors cannot occur. However, there are some applications, where bi-static systems cannot be used, e.g., using wave guide structures instead of antennas in free space. Furthermore, bi-static systems show disadvantages for high distance resolution sensors with extremely short distance operation. Since the TX and the RX antenna are placed at a certain distance, the phase response of the target cannot be mapped on an equivalent distance between the phase center of one antenna and the target, as holds true for mono-static systems. For near-field operation, a non-linear dependency between distance and phase can be observed, which is hard to linearize in a bi-static concept due to the mutual near-field interaction of both antennas and the target.

For mono-static system approaches, careful microwave circuit design is a necessary prerequisite to ensure offset minimization and a high distance resolution. Concepts for isolation-enhanced radar couplers are essential [55]. Furthermore, the matching of any component, vias and the antenna in the common TX/RX path has to be maximized.

Besides these microwave design aspects, also an active compensation of the remaining parasitic sum signal can be implemented. For this purpose, a portion of the TX signal is intentionally coupled to the RX path via a vector modulator that is formed by a 2π phase shifter and a variable attenuator. Using an appropriate control algorithm for this vector modulator, the decoupled TX portion can be phase shifted and scaled in a way leading to destructive interference with the parasitic signal in the RX path. Extensive cancellation of the static DC offset can be achieved.

### 4.2. Dynamic Offset

Besides the static offset, also dynamic parasitic components can be observed in the RX signal. In most cases, they are resulting from the interaction of the environment with the six-port interferometer, like temperature effects or aging for very slow drifts, as well as multi-path or multi-target scenarios for the radar channel. Like any other CW or FSKradar, a six-port interferometer is not able to distinguish between different targets, as well. This problem occurs if several targets are present inside the main lobe of the interferometer’s antenna. The backscattered signal of any target will lead to a sum signal in the RX path featuring the same frequency as the TX signal, but an average phase resulting from the respective phases of any target weighted by the respective reflected signal power. The six-port interferometer will display exactly one, but the wrong phase measurement result. The original response of the target is not retrievable for any CW radar architecture, including the six-port interferometer. The same effect holds true for a one-target scenario with additional multi-path influences.

If any parasitic reflector inside the antenna beam in addition to the target is in a fixed position, the resulting offset will be static. In most cases, where these undesired multi-targets are formed by mounting structures, covers, etc., the scenario will stay fixed for a long time. Therefore, the multi-target influence can be reduced by the same vector modulator that is also used to compensate the static offset as described above. Multi-path interference is varying over distance, velocity and time; it is hard to estimate and separate from the target signal. The only option may be a reference sweep of the target and linearizing the measured values in a post-processing stage. However, such calibration movement of the target is not feasible for many practical applications. For this reason, multi-path influences must be avoided by specifying the radar channel for minimum multi-path reflections and using high gain antennas. The dynamic offset is illustrated in the lower plots of Figure 18 in azure, as the remaining deviation after offset, gain and phase correction.

### 4.3. I/Q Impairments

Besides parasitic offsets, also the tolerances of any front-end component will degrade the six-port interferometer’s dynamic range and linearity. From the baseband point of view, these non-idealities will distort the intentional differential *I/Q* representation of the six-port’s output signals. Therefore, the *I/Q* mismatch is estimated from the current values and equalized to linearize the complete system. Since the phase difference of a moving target will shift over 2π for a traveled distance of half the wavelength, all acquired data must be placed on a circle in the complex valued *I/Q* plane. Therefore, the gain errors of the in-phase and quadrature signal, as well as their phase error have to be corrected as depicted in the lower plots of Figure 18. For a first approximation, the change in the free space loss of the backscattered signal for short distance changes up to some meters can be neglected in most cases. Due to any of the non-idealities, the ideal *I/Q* circle is distorted to an ellipse that is also shifted from the point of origin of the *I/Q* plane. Phase errors for each baseband voltage can be derived and used for the control algorithms of the compensation implementations by calculating the differences from the ideal unity circle gain. One approach of this *I/Q* equalizer will be presented in the following section.

### 4.4. Linearization and Compensation Techniques

Non-ideal influences in any stage of the circuitry have to be estimated and compensated for achieving maximum performance. Some of the impairments may be calibrated after manufacturing; some have to be estimated during operation. The following section discusses compensation strategies for the most important non-ideal effects.

#### 4.4.1. *I/Q* Equalization

For a moving target in a real measurement (index *m*) setup, the I/Q signals from Equations (10) and (11) are distorted and can be written as [56]:

(19)Im=ℜ{Z_m}=AIcosφz(t)+OI,(20)Qm=ℑ{Z_m}=AQsinφz(t)+φQ+OQ

All static non-idealities of the six-port front-end, except distortions due to the detectors, will result in either amplitude errors ( $A_{Q}/A_{I}\neq 1$ ), phase imbalances ( $\varphi_{Q}\neq 0$ ) or an offset ( $O_{I}\neq 0$ , $O_{Q}\neq 0$ ). Equations (10) and (11) describe an ideal circle in the complex plane, whereas Equations (19) and (20) represent in general an ellipse.

For a marginal phase error ( $\varphi_{Q}\approx 0$ ), a normalization of the baseband or I/Q signals would be sufficient. However, there is a major disadvantage of this method: an exact normalization would require the measurement of the maximum and the minimum signal values. This can be either achieved by a large number of measured positions or by measuring well-known positions (i.e., with the maximum and minimum values) during an initial calibration step.

Using Equations (19) and (20), with a minimum of five measurement points for distinct, possibly unknown, positions of the target, an ellipse can be fitted into the data. However, for a more robust calibration, more than five distinct measurement points are preferable. Various fitting methods are known from pattern recognition and communication technology research groups. After the reconstruction of the ellipse, parameters for the I/Q equalization can be extracted. This approach is also known from mixer-based *I/Q* radar systems [57].

**Figure 19:**
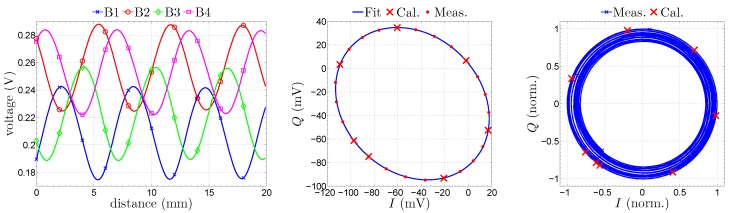
Measured baseband voltages, I/Q data and after equalization (from left to right).

Figure 19 shows on the left the distorted measured baseband signals with different offsets and amplitude imbalances, as well as a minor deviation from the π/2 spacing between the baseband signals. After calculating the I/Q signals (sub-figure in the middle), seven distinct data points at unknown positions have been selected and applied to the least square fitting [56] of an ellipse. It can be clearly noticed in Figure 19 that the additional measurement data (red points) are exactly located on the fitted ellipse. After an equalization, i.e., the mapping of the elliptical data onto the unit circle in the complex plane (sub-figure on the right), the movement of the target can be accurately calculated.

By this approach, accuracies of ±70μm at a distance of 1 m can be achieved. The remaining error can be mainly traced back to the detectors and their deviation from the pre-assumed square-law operation. Therefore, a detector linearization is often required previously to the I/Q equalization. For measurements within the antenna’s near field or in strongly-absorbing materials, the shown algorithm has to be extended to a spiral fitting and equalization [58].

#### 4.4.2. Offset Compensation

As discussed in Section 4.1 and Section 4.2, several non-ideal effects will result in offsets for the four output signals. These offsets will inherently be eliminated by the shown ellipse equalization in the digital baseband circuitry, but in the analog domain, these offsets will still exist, which limits the ADCs’ dynamic range. Even with baseband amplifiers, the full dynamic range cannot be achieved. As mentioned in Section 2.2, various compensation structures can be utilized in the RF path, e.g., an AGC or an offset compensation circuitry, which cancel the static DC offsets extensively.

The key part in analog compensation is to equalize the RF input power of both input ports of the six-port structure by two variable attenuators in the reference path and the receive path, respectively, since maximum phase sensitivity is ensured for maximum destructive interference for counter-phase superposition. If both input signals exhibit the same power, perfect cancellation of both signals occurs. The control algorithm for the attenuators at the six-port’s input ports will set the reference and the receive signal to nearly equal power level, but not exactly the same. This is based on the fact that for total cancellation, deviations in the distance may be hidden in noise. A slight difference in the signals’ power will result in a small offset, ensuring that the lower turning point will still be above the noise floor, while still operating the analog baseband with a high dynamic range.

For setting the discussed AGC for power equalization of the input signals, one has to observe another important signal component of the static DC offset in addition. Especially for higher operating frequencies, a portion of the transmit signal is reflected at the feeding point of the antenna due to limited matching or parasitically coupled in the receive path. Since this parasitic transmit power may be orders of magnitude higher than the received signal scattered back by the target, the orthogonality for each output path may be distorted by this static signal. This impairment may be directly compensated in the RF domain by estimating the parasitic transmit signal portion in value and phase for the receive path and coupling an equivalent portion of the transmit signal in counter phase to the receive path, canceling out the parasitic signal. The compensation circuitry is realized by a vector modulator and directional couplers. The required variable attenuator, as well as the variable phase shifter have to be set recursively, due to the mutual interaction on both stages, with an adjustable step size by an intelligent compensation algorithm for a fast and effective leakage cancellation or equalization. For ideal compensation, the receive signal will only consist of the receive signal scattered back by the target, which leads to an optimal usage of the ADCs’ dynamic range.

One has to keep in mind that if the AGC modifies its corresponding attenuators, the *I/Q* offset and eventually the ellipse form will be changed, as well, due to mutual variation of the phase and value for each tunable component for which reason, the offset compensation algorithm, as well as the ellipse reconstruction have to be rerun afterwards.

Furthermore, instead of using four single-ended ADCs, two differential ones can be considered for a direct *I/Q* signal output. Using a differential conversion simplifies the analog offset compensation as the DC offsets do not have to be extinguished, but equalized for each differential baseband signal pair.

#### 4.4.3. Detector Linearization

Six-port theory regards the diode circuitry at each output port as ideal power detectors. This holds only true for low and medium RF signal power operating the detectors in their square law region [59], i.e., approximately quadratic dependency between input power and output voltage. In reality, the transfer characteristic for a wide range of input powers follows exponential instead of quadratic behavior. This leads to a non-linear mapping of power to voltage and results in non-linear phase behavior [60,61]. This impairment may be compensated if the real characteristic of each detector is known during operation. Therefore, for dedicated calibration cycles during operation, the transfer characteristic of each detector is calibrated by an RF power sweep [62]. For this purpose, an additional switch is inserted in the receive path for only supplying the reference signal to the six-port. From Equations (1)–(4), one will find that eliminating the receive signal $S_{2}$ will result in a well-defined distribution of the signal $S_{1}$ to the detectors. While sweeping the reference signal’s power by the variable attenuator for the offset compensation from the previous section to a set of dedicated values, the equivalent DC voltage is tracked at the detectors’ output port and stored for equalizing the detectors’ characteristic during operation.

## 5. Discussion

### 5.1. The Six-Port Concept’s Benefits and Drawbacks

The main advantages of the six-port interferometer are its low complexity hardware setup and its extraordinary phase resolution. The latter is based on the fact that compared to mixer-based *I/Q* receivers, the *I/Q* generation and down conversion are split into separate stages: the passive six-port structure will interferometrically generate the *I/Q* representation directly in the RF domain. There is neither noisy circuitry nor non-linear processing stages involved, resulting in strictly linear behavior. The resulting differential *I/Q* signals are then brought to the baseband in a subsequent step by diode detectors, in the best case operating in their square law region and not spreading the signal’s energy to harmonic components with significant portions of power.

Like most other metrology technologies, the six-port interferometer is only able to deliver its full performance if non-ideal effects are estimated and compensated in situ during operation. This is realized by specific algorithms, in some cases accompanied by additional hardware building blocks. The basic concept can be found in [63]. The biggest impairment components result from an undesired DC offset due to limited matching of the circuit elements in the transmit path, as well as the limited isolation between the TX and RX signal. A large offset reduction can be achieved by careful system design, e.g., enhanced matching and isolation [55] or by choosing a bi-static instead of a mono-static concept, if applicable. The influence of the remaining offset can be reduced by estimating the current characteristics of each power detector and using this knowledge for compensating the non-linearity in the power-to-voltage conversion [62]. Additionally, fabrication tolerances of the complete front-end will lead to a further distortion of the four baseband output voltages of the six-port system, which form a balanced representation of a complex valued number. This distortion can be estimated from the deviation of the ideal unity circle in the complex Gaussian plane, leading to compensation coefficients for equalizing each baseband signal in the digital domain. This procedure can be conducted both as initial calibration after manufacturing [64] and in situ during operation [56]. Furthermore, the baseband processing platform has to fit the requirements defined by the range resolution and the accuracy of the total system [65]. In this context, the number of bits per processing step or the strategy for efficient implementation of certain algorithmic components has to be considered from the processing time and memory point of view [66].

One has to keep in mind two further peculiarities of a six-port interferometer.

#### 5.1.1. Ambiguities

Like all interferometric or phase-based CW concepts, the six-port exhibits the drawback of a measurement ambiguity, which directly results from the pivotal Equation (13): due to the arctan-function, the phase values will be unambiguous for a limited phase interval of [−π;π] , which leads to an unambiguous distance measurement range of only $\frac{\lambda }{2}$ .

However, this ambiguity can be resolved using various methods. The easiest way would be to just monitor the target, count the recurring phase jumps and unwrap the phase. This is a very simple approach, but it needs permanent surveillance of the target and knowledge of the starting position. Therefore, this should only be used for measurement tasks, where the knowledge of the relative distance change is sufficient, e.g., vibration monitoring.

**Figure 20:**
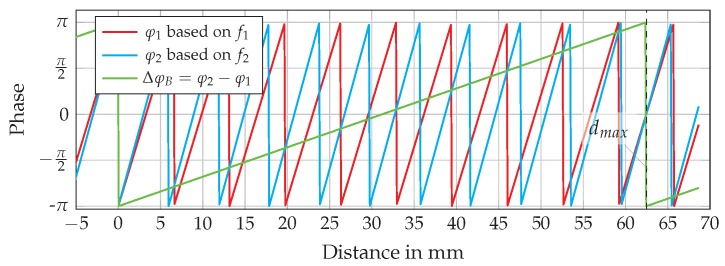
Phase jumps due to six-port-related unambiguity at CW and FSK setup.

A second approach is the usage of an FMCW modulation of the radar signal in certain time steps to determine the coarse, due to limited bandwidth, absolute distance of the target before switching back to CW mode for interferometric phase evaluation. With this coarse absolute distance, an accurate absolute distance value can be evaluated by only using the CW-derived phase evaluation by adding an offset corresponding to the period of the ambiguity calculated from the coarse estimation. Nevertheless, the resolution of common band-limited FMCW systems is often not high enough to ensure the required distance accuracy of $\pm \frac{\lambda }{4}$ . Therefore, an alternative approach is to use a second single CW tone featuring a slightly different frequency, as described in [67]. By this setup, the unambiguous range can be extended to half of the wavelength of the frequency difference (the beat frequency) of the single tones. This behavior is shown in Figure 20, where the phases of two single tones around 24 GHz with a spacing of 2.4 GHz and the resulting phase of the beat frequency are shown. It is obvious from the periodicity of the beat signal that the unambiguous range is extended from about 6 mm for the single tone evaluation of $f_{1}$ or $f_{2}$ up to 60 mm. This range extension depends on the selected frequency spacing and is limited to a few meters, which is discussed in [68].

#### 5.1.2. Multi-Path and Multi-Target Effects

From general radar theory, it is well known that a separation of targets, as well as detecting the effects of several independent propagation paths for the radar signal are only possible by spending a minimum amount of bandwidth [1]. Consequently, CW-based six-port interferometry is not capable of distinguishing either several targets or multiple paths. For a multi-target environment, the system will deliver one phase value consisting of all distances of targets inside the radar beam weighted by their radar cross-section. This leads to the interesting fact that multi-target environments may also be suitable for six-port metrology if only the desired target is moving and all other scatterers in the signal path remain static at their fixed position. This will lead to a static DC offset in the received signal superimposed to the phase shift induced by the moving wanted target. The static offset may be compensated in the digital domain by the algorithms discussed in Section 4.4.1. Nevertheless, optimum system performance will be reached by ensuring a scattering and multi-path free observation channel. This restriction may be relaxed if high gain antennas may be used resulting in a tight focusing and suppressing influences from outside the main lobe.

#### 5.1.3. Current Challenges in Six-Port Research

In the context of precise remote distance measurements in industrial metrology, calibration of the sensing unit is always a challenge. This holds also true for six-port interferometry [69]. To derive micrometer range resolution, an extremely linear phase performance over power and frequency is required. During operation in the industrial scenario, it is impossible to calibrate the system by using dedicated calibration standards [70] or precise reference positions of the target [64]. Therefore, one important focus of the current research in six-port interferometry is blind estimation of the non-idealities to derive linearization coefficients for phase compensation. One of the most dominant components for non-ideal phase behavior is the power detector resulting from the fact that the diodes’ characteristic drift over temperature and devices and shows only for a small region a more or less quadratic behavior. Current research addresses this issue in several ways: By careful and adaptive system design, it is tried to hold the input signal level of the detectors in a small range. Another approach tries to estimate the actual characteristic of each individual detector in situ and to equalize the non-idealities in the digital domain. Apart from that topic, any component of the interferometric six-port system shows potential for enhancements [11,71,72]. The majority of papers is currently investigating the use of six-port interferometry for a variety of measurement applications [73,74]. However, since, six-port interferometry for metrology is a niche topic. Most research groups focus on the use of the six-port concept as a receiver or even the transceiver for communications [75,76].

### 5.2. Comparison to State-Of-The-Art Industrial Remote Metrology

There is a wide range of scientific publications for common sensing principles, which focuses on very specific details, respectively, and only giving specific values for the optimized criterion. Thus, a general and fair conclusion is hard to draw. Hering et al. present in [77] a summary of professional distance and ranging sensors giving typical values for commercially available sensors, which cover about 80% of the market according to the authors. This table is reproduced in Table 1 as a base for comparing these common metrology approaches to radar sensors and six-port interferometry. The measurement value update rate was not available for some concepts in the source table and has been cited from exemplary product manuals.

Compared to conventional industrial remote distance metrology, radar in general and especially six-port technology show some advantages, e.g., optical- or laser-based systems exhibit reliability issues in harsh environments featuring dust, fog, glowing surfaces or bright illumination. Ultrasonic distance metrology shows comparably low measurement value update rates due to the lower propagation velocity of the acoustic wave. Inductive, as well as capacitive distance sensors are only suitable for ultra-short range metrology. The six-port interferometer is able to cope with all of these preconditions making this approach a promising candidate for supplementing state-of-the-art industrial metrology technologies.

For the common radar principles, a comparison for industrial applications exhibiting one-dimensional distance measurements in a one target scenario has been conducted, as well. Their characteristic performances can be found in Table 2. One of the key parameters, the measurement value update rate, is rarely defined for industrial radar systems. In most scientific publications, only the sweep time for the frequency ramp is given. The delay due to all further signal processing steps is neglected in most cases. To be nevertheless able to compare common radar systems to six-port interferometry, the cycle time of the field bus interface for the different architectures will be used as a measure for the measurement value update rate.

CW radar and six-port interferometry will have the same update rate performance, since both concepts differ only in their building blocks and use the same physical methodology for distance analysis. For the comparison of pulse and FMCW radar to six-port interferometry, commercially available radar sensors are used as a benchmark and cited in Table 2.

For CW and FSK distance sensors, no commercially available systems were found, only RF building blocks, e.g., [86]. Furthermore the stated applications focus in nearly all cases on Doppler velocity evaluation and not on distance sensing, e.g., for [87,88]. Therefore, there are no entries for CW and FSK radars in Table 2. From empirical investigations of the author and his group on CW radar front-ends with the same hardware complexity level as for a six-port interferometer, the distance resolution, as well as the precision in most cases are slightly, in some cases up to one order of magnitude declined, compared to a respective six-port setup. The reason is discussed in the following.

The six-port interferometer belongs to the class of microwave sensing technologies, and its architecture is very similar to a CW radar [89]. Its superior range resolution results from the difference in the operation: the formation of complex valued baseband output voltages is split for the six-port front-end to a phase shifted superposition of the input signals in the microwave domain by the help of the passive six-port structure, which itself is formed by microwave couplers. The resulting four microwave signals are scaled in their respective power proportional to the relative and unknown phase difference between the two input signals. The desired measurement information, e.g., the distance to the target, is derived in the baseband after the down-conversion of the microwave to voltage signals by power detectors, which ideally feature a dominant and undistorted quadratic dependency for this conversion. Common CW radar relies on active multiplicative mixing, which combines these steps in the mixing process. This may lead to limited phase accuracy due to non-linear effects in the active stages. If the power of both input signals is controlled carefully, the six-port interferometer’s phase resolution directly connected to the range resolution will exceed the one for common CW radar. Besides free space distance measurements and direction finding, also mechanical strain analysis has been addressed in this publication. Due to the high measurement value update rate, also vibrations and pulse-like movements can be detected by fast and precise consecutive distance measurements. FMCW radar is able to separate several targets and is suitable in multi-path environment. This concept requires solving the FFT of the baseband signals, leading to lower measurement value update rates compared to the six-port interferometer. Compared to industrial pulse radar, the six-port architecture is also suitable for short range applications and will result in higher range resolution in most scenarios.

## 6. Conclusions

This paper gives a review of six-port technology for industrial metrology. By discussing the building blocks, it shows that this architecture features low complexity combined with precise and accurate measurements. The applicability to distance, alignment and mechanical strain measurements has been presented. Furthermore, limitations and non-ideal behavior, as well as compensation and linearization techniques have been shown. By means of the distance measurement as an example, it is concluded from a comparison to other state-of-the-art industrial metrology approaches that six-port technology may bridge the gap in technology and is an interesting tool in this context.

## Figures and Tables

**Table 1:** Typical values for commercially available sensors for distance measurements and ranging according to [77] if not stated otherwise.

Principle	Typical Measurement Range	Precision	Measurement Value Update Rate
Inductive	0.1 mm to 50 mm	100 µm to 50 µm	1 kHz
Capacitive	0 mm to 10 mm	100 µm to 50 µm	100 Hz
Optical Triangulation	45 mm to 85 mm [78]	20 µm ^(1)^ [78]	40 Hz [78]
Optical Time-of-Flight	200 mm to 6000 mm	±15 mm [79]	40 Hz [79]
Optical Interferometry	0 mm to 4000 mm	1 µm [80]	100 MHz [80]
Ultrasonic	250 mm to 4000 mm	1 mm to 500 µm	15 Hz to 250 Hz [81]
Magnetostrictive	25 mm to 2000 mm	50 µm to 10 µm	1.5 kHz [82]

^(1)^ No precision information listed in the product manual; the given value states the resolution.

**Table 2:** Typical values for commercially available radar sensors for distance measurements and ranging taken from representative product manuals (see the citations).

Principle	Frequency	Typical Measurement Range	Precision	Measurement Value Update Rate	Citation
Pulse	25 GHz	0.21 m to 13 m	10 mm ^(1)^	0.5 Hz	[83]
FMCW	24 GHz to 26 GHz	0.2 m to 80 m	±3 mm	10 Hz ^(2)^	[84]
six-port	24 GHz	0.01 m to 2.0 m ^(3)^	±40 µm	20 Hz ^(4)^	[85]

^(1)^ No precision stated in the manual; the given value is the maximum measurement error; ^(2)^ No measurement value update rate stated; frequency results from the serial interface; ^(3)^ Only evaluated up to 2 m due to distance limitations in laboratory; ^(4)^ Limited by the utilized serial data interface.

## References

[1] Jaeschke T., Bredendiek C., Kuppers S., Pohl N. (2014). High-Precision D-Band FMCW-Radar Sensor Based on a Wideband SiGe-Transceiver MMIC. IEEE Trans. Microw. Theory Tech..

[2] Engen G.F. (1969). An Introduction to the Description and Evaluation of Microwave Systems Using Terminal Invariant Parameters.

[3] Engen G. (1977). The six-port Reflectometer: An Alternative Network Analyzer. IEEE Trans. Microw. Theory Tech..

[4] Engen G. (1997). A (Historical) Review of the six-port Measurement Technique. IEEE Trans. Microw. Theory Tech..

[5] Ghannouchi F.M., Mohammadi A. (2009). The Six-Port Technique.

[6] Caron M., Akyel A., Ghannouchi F.M. (1995). A Versatile Easy to Do six-port Based High Power Reflectometer. J. Microw. Power Electromagn. Energy.

[7] De Souza Rolim A.L., Belfort de Oliveira A.J., de Melo M.T. Six-port Complex Permittivity Measurements. Proceedings of the European Microwave Conference, EuMC 2006.

[8] Wang M., Haddadi K., Glay D., Lasri T. Compact near-field microwave microscope based on the multi-port technique. Proceedings of the European Microwave Conference, EuMC 2010.

[9] Hentschel T. (2005). The six-port as a Communications Receiver. IEEE Trans. Microw. Theory Tech..

[10] Bosisio R.G., Zhao Y.Y., Xu X.Y., Abielmona S., Moldovan E., Xu Y.S., Bozzi M., Tatu S.O., Nerguizian C., Frigon J.F. (2008). New-Wave Radio. IEEE Microw. Mag..

[11] Hannachi C., Tatu S.O. A new compact V-band six-port receiver for high data-rate wireless applications. Proceedings of the 2015 IEEE Topical Conference on Wireless Sensors and Sensor Networks (WiSNet).

[12] Tatu S., Serban A., Helaoui M., Koelpin A. (2014). Multiport Technology: The New Rise of an Old Concept. IEEE Microw. Mag..

[13] Li J., Bosisio R., Wu K. A Collision Avoidance Radar Using six-port Phase/Frequency Discriminator (SPFD). Proceedings of the IEEE MTT-S International Microwave Symposium Digest.

[14] Stelzer A., Diskus C., Lubke K., Thim H. (1999). A Microwave Position Sensor with Submillimeter Accuracy. IEEE Trans. Microw. Theory Tech..

[15] Gutierrez Miguelez C., Huyart B., Bergeault E., Jallet L.P. (2000). A New Automobile Radar Based on the six-port Phase/Frequency Discriminator. IEEE Trans. Veh. Technol..

[16] Moldovan E., Tatu S.O., Gaman T., Wu K., Bosisio R.G. (2004). A New 94-GHz six-port Collision-Avoidance Radar Sensor. IEEE Trans. Microw. Theory Tech..

[17] Boukari B., Moldovan E., Affes S., Wu K., Bosisio R., Tatu S.O. Six-port FMCW collision avoidance radar sensor configurations. Proceedings of the Canadian Conference on Electrical and Computer Engineering, CCECE 2008.

[18] Haddadi K., Lasri T. V-band two-tone continuous wave radar operating in monostatic/bistatic mode. Proceedings of the 2012 9th European Radar Conference (EuRAD).

[19] Haddadi K., Lasri T. The muti-port technology for microwave sensing applications. Proceedings of the 2012 IEEE MTT-S International Microwave Symposium Digest (MTT).

[20] Haddadi K., Lasri T. Six-port technology for millimeter-wave radar and imaging applications. Proceedings of the 2014 IEEE Topical Conference on Wireless Sensors and Sensor Networks (WiSNet).

[21] Koelpin A., Vinci G., Laemmle B., Kissinger D., Weigel R. (2010). The six-port in Modern Society. Microw. Mag..

[22] Xu Y., Bosisio R.G. (1998). Effects of local oscillator leakage in digital millimetric six-port receivers (SPRs). Microw. Opt. Technol. Lett..

[23] Li J., Bosisio R., Wu K. A six-port Direct Digital Millimeter Wave Receiver. Proceedings of the IEEE MTT-S International Microwave Symposium Digest.

[24] Schiel J.C., Tatu S.O., Wu K., Bosisio R.G. Six-port Direct Digital Receiver (SPDR) and Standard Direct Receiver (SDR) Results for QPSK Modulation at High Speeds. Proceedings of the 2002 IEEE MTT-S International Microwave Symposium Digest.

[25] Luy J.F., Mueller T., Mack T., Terzis A. (2004). Configurable RF receiver architectures. IEEE Microw. Mag..

[26] Seman N., Bialkowski M., Ibrahim S., Bakar A. Design of an Integrated Correlator for Application in Ultra Wideband six-port Transceivers. Proceedings of the IEEE Antennas and Propagation Society International Symposium, APSURSI ’09.

[27] Haddadi K., Wang M.M., Loyez C., Glay D., Lasri T. (2010). Four-Port Communication Receiver With Digital IQ-Regeneration. IEEE Microw. Wirel. Compon. Lett..

[28] Koelpin A. (2010). Der Erweiterte Sechstor-Empfänger Ein Systemübergreifender Ansatz für Kommunikations-und Messaufgaben. Ph.D. Thesis.

[29] Xu X., Bosisio R.G., Wu K. (2006). Analysis and implementation of six-port software-defined radio receiver platform. IEEE Trans. Microw. Theory Tech..

[30] Akyel C., Ghannouchi F.M., Caron M. (1994). A New Design for High-Power six-port Reflectometers using Hybrid Stripline/Waveguide Technology. IEEE Trans. Instrum. Meas..

[31] Boulejfen N., Kouki A., Khouaja S., Ghannouchi F.M. A homodyne multi-port network analyzer for S parameter measurements of microwave N-port circuits/systems. Proceedings of the 1998 IEEE Asia-Pacific Conference on Circuits and Systems.

[32] Yakabe T., Ghannouchi F.M., Eid E.E., Fujii K., Yabe H. (2002). Six-port self-calibration based on active loads synthesis. IEEE Trans. Microw. Theory Tech..

[33] Tatu S.O., Cojocaru R.I., Moldovan E. Interferometric quadrature down-converter for 77 GHz automotive radar: Modeling and analysis. Proceedings of the 2010 European Radar Conference (EuRAD).

[34] Zhang H., Li L., Wu K. (2008). Software-Defined six-port Radar Technique for Precision Range Measurements. IEEE Sens. J..

[35] Xiao F., Ghannouchi F., Yakabe T. (2003). Application of a six-port wave-correlator for a very low velocity measurement using the Doppler effect. IEEE Trans. Instrum. Meas..

[36] Djerafi T., Daigle M., Boutayeb H., Zhang X., Wu K. Substrate Integrated Waveguide six-port Broadband Front-End Circuit for Millimeter-Wave Radio and Radar Systems. Proceedings of the European Microwave Conference, EuMC 2009.

[37] Vinci G., Lindner S., Barbon F., Weigel R., Koelpin A. (2012). Promise of a Better Position. Microw. Mag..

[38] Mann S., Erhardt S., Lindner S., Lurz F., Linz S., Barbon F., Weigel R., Koelpin A. Diode Detector Design for 61 GHz Substrate Integrated Waveguide six-port Radar Systems. Proceedings of the IEEE Topical Conference on Wireless Sensors and Sensor Networks (WiSNet).

[39] Xu X., Bosisio R.G., Wu K. (2005). A new six-port junction based on substrate integrated waveguide technology. IEEE Trans. Microw. Theory Tech..

[40] Mann S., Lurz F., Linz S., Lindner S., Will C., Wibbing S., Weigel R., Koelpin A. Substrate Integrated Waveguide Fed Antenna for 61 GHz Ultra-Short-Range Interferometric Radar Systems. Proceedings of the IEEE Topical Conference on Wireless Sensors and Sensor Networks (WiSNet).

[41] Garcia B.A., Kerneves D., Huyart B. Measurement of Direction of Arrival for Radar Application. Proceedings of the 32nd European Microwave Conference.

[42] Vinci G., Barbon F., Weigel R., Koelpin A. A Novel, Wide Angle, High Resolution Direction-Of-Arrival Detector. Proceedings of the European Microwave Week (EuMW).

[43] Koelpin A., Vinci G., Laemmle B., Lindner S., Barbon F., Weigel R. (2012). Six-port Technology for Traffic Safety. IEEE Microw. Mag..

[44] Buff W., Klett S., Rusko M., Ehrenpfordt J., Goroli M. (1998). Passive remote sensing for temperature and pressure using SAW resonator devices. IEEE Trans. Ultrason. Ferroelectr. Freq. Control.

[45] Pohl A., Ostermayer G., Seifert F. (1998). Wireless sensing using oscillator circuits locked to remote high-Q SAW resonators. IEEE Trans. Ultrason. Ferroelectr. Freq. Control.

[46] Beckley J., Kalinin V., Lee M., Voliansky K. Non-contact torque sensors based on SAW resonators. Proceedings of the IEEE International Frequency Control Symposium and PDA Exhibition.

[47] Hamsch M., Hoffmann R., Buff W., Binhack M., Klett S. (2004). An interrogation unit for passive wireless SAW sensors based on Fourier transform. IEEE Trans. Ultrason. Ferroelectr. Freq. Control.

[48] Kalinin V., Beckley J., Makeev I. High-speed reader for wireless resonant SAW sensors. Proceedings of the European Frequency and Time Forum (EFTF).

[49] Lurz F., Lindner S., Mann S., Linz S., Weigel R., Koelpin A. Precise and Fast Frequency Determination of Resonant SAW Sensors by a Low-Cost six-port Interferometer. Proceedings of the IEEE International Instrumentation and Measurement Technology Conference (I2MTC).

[50] East P. (2012). Fifty years of instantaneous frequency measurement. Radar Sonar Navig. IET.

[51] Buff W., Rusko M., Vandahl T., Goroll M., Moller F. A differential measurement SAW device for passive remote sensoring. Proceedings of the Ultrasonics Symposium.

[52] Lurz F., Lindner S., Mann S., Linz S., Barbon F., Weigel R., Koelpin A. A Low-Cost 2.4 GHz Frequency Measurement System for Microsecond Time Domain Pulses Based on six-port Technology. Proceedings of the 44th European Microwave Week, EuMA.

[53] Mailand M., Richter R. Blind IQ-Regeneration for six-port-Based Direct Conversion Receiver with Low Analog Complexity. Proceedings of the 11th European Wireless Conference 2005–Next Generation Wireless and Mobile Communications and Services (European Wireless).

[54] Park B.K., Boric-Lubecke O., Lubecke V.M. (2007). Arctangent Demodulation With DC Offset Compensation in Quadrature Doppler Radar Receiver Systems. IEEE Trans. Microw. Theory Tech..

[55] Mann S., Vinci G., Lindner S., Linz S., Barbon F., Weigel R., Koelpin A. 61 GHz six-port Radar Frontend for High Accuracy Range Detection Applications. Proceedings of the IEEE-APS Topical Conference on Antennas and Propagation in Wireless Communications.

[56] Linz S., Vinci G., Lindner S., Mann S., Lurz F., Barbon F., Weigel R., Koelpin A. *I/Q* Imbalance Compensation for six-port Interferometers in Radar Applications. Proceedings of the 2014 IEEE European Microwave Conference (EuMC).

[57] Singh A., Gao X., Yavari E., Zakrzewski M., Cao X.H., Lubecke V., Boric-Lubecke O. (2013). Data-based quadrature imbalance compensation for a CW Doppler radar system. IEEE Trans. Microw. Theory Tech..

[58] Sporer M., Lurz F., Schluecker E., Weigel R., Koelpin A. Underwater Interferometric Radar Sensor for Distance and Vibration Measurement. Proceedings of the IEEE Topical Conference on Wireless Sensors and Sensor Networks (WiSNet), Michael Sporer.

[59] Hoer C.A., Roe K.C., Allred C.M. (1976). Measuring and minimizing diode detector nonlinearity. IEEE Trans. Instrum. Meas..

[60] Colef G., Karmel P.R., Ettenberg M. (1990). New in-situ calibration of diode detectors used in six-port network analyzers. IEEE Trans. Instrum. Meas..

[61] Juroshek J.R., Hoer C.A. (1984). A Dual six-port Network Analyzer Using Diode Detectors. IEEE Trans. Microw. Theory Tech..

[62] Barbon F., Lindner S., Mann S., Linz S., Weigel R., Koelpin A. Fast In-Situ Diode Detector Characterization for six-port Interferometer Receivers. Proceedings of the 2014 IEEE Radio and Wireless Week (RWW 2014).

[63] Koelpin A., Linz S., Barbon F., Lindner S., Mann S., Lurz F., Weigel R. Selftest Strategies for Microwave Interferometry for High-Precision Industrial Distance Measurements. Proceedings of the 20th International Conference on Microwaves, Radar, and Wireless Communications.

[64] Staszek K., Linz S., Lurz F., Mann S., Weigel R., Koelpin A. Improved Calibration Procedure for six-port Based Precise Displacement Measurements. Proceedings of the IEEE Topical Conference on Wireless Sensors and Sensor Networks (WiSNet).

[65] Lindner S., Barbon F., Linz S., Lurz F., Mann S., Weigel R., Koelpin A. ADC Depending Limitations for six-port Based Distance Measurement Systems. Proceedings of the IEEE Topical Conference on Wireless Sensors and Sensor Networks 2015 (WiSNet).

[66] Barbon F., Vinci G., Lindner S., Weigel R., Koelpin A. Signal Processing Strategies for six-port Based Direction of Arrival Detector Systems. Proceedings of the 2012 9th International Multi-Conference on Systems, Signals and Devices (SSD).

[67] Lindner S., Barbon F., Mann S., Vinci G., Weigel R., Koelpin A. Dual tone approach for unambiguous six-port based interferometric distance measurements. Proceedings of the IEEE MTT-S International Microwave Symposium.

[68] Lindner S., Barbon F., Linz S., Mann S., Weigel R., Koelpin A. (2015). Distance measurements and limitations based on guided wave 24GHz dual tone six-port radar. Int. J. Microw. Wirel. Technol..

[69] Olopade A.O., Helaoui M. Mitigation of distortion and memory effect in a concurrent dual-band six port receiver. Proceedings of the 2014 44th European Microwave Conference (EuMC).

[70] Judah S.R., Holmes W. A novel sixport calibration incorporating diode detector non-linearity. Proceedings of the IEEE Conference Proceedings Instrumentation and Measurement Technology Conference.

[71] Moldovan E., Tatu S.O. Design and characterization of novel W-band wide-band couplers and six-port circuit. Proceedings of the 2015 European Microwave Conference (EuMC).

[72] Haddadi K., Loyez C. 65 nm SOI CMOS 60 GHz passive mixer for six-port technology. Proceedings of the 2016 IEEE Topical Conference on Wireless Sensors and Sensor Networks (WiSNet).

[73] Chioukh L., Djerafi T., Deslandes D., Wu K. Improvements of cardiopulmonary monitoring using harmonic six-port radar system. Proceedings of the 2015 Global Symposium On Millimeter Waves (GSMM).

[74] Haddadi K., Lasri T. Forward V-band vector network analyzer based on a modified six-port technique. Proceedings of the 2015 IEEE Topical Conference on Wireless Sensors and Sensor Networks (WiSNet).

[75] Hasan A., Helaoui M., Boulejfen N., Ghannouchi F.M. Six-port technology for MIMO and cognitive radio receiver applications. Proceedings of the 2015 IEEE Topical Conference on Wireless Sensors and Sensor Networks (WiSNet).

[76] Oesth J., Karlsson M., Serban A., Gong S. (2015). A Comparative Study of Single-Ended vs. Differential six-port Modulators for Wireless Communications. IEEE Trans. Circ. Syst. I Regul. Pap..

[77] Hering E., Schoenfelder G. (2012). Sensoren in Wissenschaft und Technik.

[78] (2016). Photoelectric Distance Sensor.

[79] (2016). Distance Sensor.

[80] (2014). Statuspro μLine F1 Laser Interferometer.

[81] (2016). Ultrasonic Sensor.

[82] (2016). NOVOSTRICTIVE Transducer up to 4250 mm touchless series TP1.

[83] (2016). SITRANS LR260 (PROFIBUS PA).

[84] (2016). 24 GHz Non-contact Radar (FMCW) Level Meter Optiwave 7300C.

[85] Linz S., Vinci G., Lindner S., Mann S., Barbon F., Weigel R., Koelpin A. (2013). A Compact, Versatile six-port Radar Sensor for Industrial and Medical Applications. IEEE Sens..

[86] InnoSenT (2016). IVS-948.

[87] InnoSenT (2016). IPM-165 Low-Cost Radar Transceiver.

[88] InnoSenT (2016). iSYS-4001-Systems.

[89] Koelpin A., Weigel R. Six-port Based Direction Finding and Ranging. Proceedings of the 20th International Conference on Microwaves, Radar, and Wireless Communications.

